# Biases in the perception of self-motion during whole-body acceleration and deceleration

**DOI:** 10.3389/fnint.2013.00090

**Published:** 2013-12-16

**Authors:** Luc Tremblay, Andrew Kennedy, Dany Paleressompoulle, Liliane Borel, Laurence Mouchnino, Jean Blouin

**Affiliations:** ^1^Faculty of Kinesiology and Physical Education, University of TorontoToronto, ON, Canada; ^2^Fédération de Recherche 3C Comportement-Cerveau-Cognition, Centre National de la Recherche Scientifique – Aix-Marseille UniversityMarseille, France; ^3^Laboratoire de Neurosciences Intégratives et Adaptatives, Centre National de la Recherche Scientifique – Aix-Marseille UniversityMarseille, France; ^4^Laboratoire de Neurosciences Cognitives, Centre National de la Recherche Scientifique – Aix-Marseille UniversityMarseille, France

**Keywords:** vestibular, perception, body rotation, head rotation, passive motion, acceleration, deceleration, velocity storage

## Abstract

Several studies have investigated whether vestibular signals can be processed to determine the magnitude of passive body motions. Many of them required subjects to report their perceived displacements offline, i.e., after being submitted to passive displacements. Here, we used a protocol that allowed us to complement these results by asking subjects to report their introspective estimation of their displacement continuously, i.e., during the ongoing body rotation. To this end, participants rotated the handle of a manipulandum around a vertical axis to indicate their perceived change of angular position in space at the same time as they were passively rotated in the dark. The rotation acceleration (Acc) and deceleration (Dec) lasted either 1.5 s (peak of 60°/s^2^, referred to as being “High”) or 3 s (peak of 33°/s^2^, referred to as being “Low”). The participants were rotated either counter-clockwise or clockwise, and all combinations of acceleration and deceleration were tested (i.e., AccLow-DecLow; AccLow-DecHigh; AccHigh-DecLow; AccHigh-DecHigh). The participants’ perception of body rotation was assessed by computing the gain, i.e., ratio between the amplitude of the perceived rotations (as measured by the rotating manipulandum’s handle) and the amplitude of the actual chair rotations. The gain was measured at the end of the rotations, and was also computed separately for the acceleration and deceleration phases. Three salient findings resulted from this experiment: (i) the gain was much greater during body acceleration than during body deceleration, (ii) the gain was greater during High compared to Low accelerations and (iii) the gain measured during the deceleration was influenced by the preceding acceleration (i.e., Low or High). These different effects of the angular stimuli on the perception of body motion can be interpreted in relation to the consequences of body acceleration and deceleration on the vestibular system and on higher-order cognitive processes.

## INTRODUCTION

The study of space perception and navigation has devoted a great deal of attention to the role of the vestibular information. One explanation for this focus is the fact that the labyrinths of the inner ear provide information about the linear and angular displacements of the head relative to space and also of the body by combining vestibular and neck muscle proprioception inputs. For comparison, the visual inputs, in their preliminary stage of processing, are more ambiguous because a given change of retinal inputs can result from either self motion (i.e., head and/or body) or motion of the objects from the environment.

Psychophysical studies have indisputably demonstrated that one can process vestibular information to create a percept of self-motion in space. Among the most convincing demonstrations for the importance of vestibular output is the much larger motion perception threshold for both rotation directions after total bilateral vestibular ablation (Valko et al., 2012) or for rotations toward the lesion side after unilateral vestibular loss (Cousins et al., 2013). In addition, Fitzpatrick et al. (2002) reported that galvanic stimulation of the vestibular system (GVS) in neurologically intact individuals changes their perception of actual body rotations. These authors found that motion perception is enhanced when both the GVS and the rotation are congruent (i.e., when both stimuli activate and inhibit the same labyrinths’ side) and is attenuated when both stimulations are incongruent.

**FIGURE 1:**
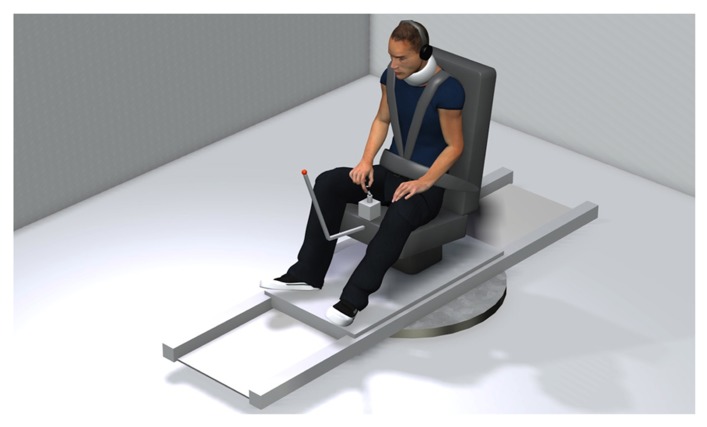
**Schematic representation of the experimental apparatus**.

The goal of the present study was to specifically assess the cognitive estimate of body displacement during the course of discrete body rotations. Previous investigations on motion perception during ongoing body displacements have essentially used protocols where subjects were asked to adjust the speed of a hand-steered lever according to the perceived rotation intensity (Guedry, 1974; Okada et al., 1999; Seemungal et al., 2007; Sinha et al., 2008; Bertolini et al., 2012; Shaikh et al., 2013) or to continuously point at a remote (unseen) target during body displacement (e.g., Loomis et al., 1992; Ivanenko et al., 1997a,b; Philbeck et al., 2001; Bresciani et al., 2005; Guillaud et al., 2006; Blouin et al., 2010; Frissen et al., 2011). Because the former methods essentially test whether the subjects perceive the rotation as increasing, decreasing or constant, it does not provide information regarding the actual perception of angular displacement (Shaikh et al., 2013). On the other hand, with the latter methods (i.e., continuous pointing), one cannot determine the degree to which the responses provided by the subjects are issued from vestibular-issued sensorimomotor processes or from introspective estimation of body displacement. Indeed, experimental evidence was given that the compensatory arm movements leading to such hand stabilization derive from vestibulomotor transformations that involve negligible cognitive processes (Bresciani et al., 2005; Blouin et al., 2010). Moreover, the fact that the motor systems may have access to spatial information that may not be readily available to the perceptual system (Goodale and Milner, 1992; Prablanc and Martin, 1992; Milner and Goodale, 2008) also increases the uncertainty regarding the contribution of perceptual processes in stabilizing the hand during body motion. In the present study, participants were asked to indicate, by rotating the handle of a manipulandum mounted on a vertical axis (see **Figure 1**), their perceived change of angular position in space during passive body rotations. With this method, an unbiased response would be associated with rotating the manipulandum’s handle in the same direction and amplitude as the actual body rotation.

When consulting the extant literature, the relatively good accuracy with which subjects perceive their passive motions could be viewed as odd considering the effects of these motions on the labyrinths during their deceleration phase. Indeed, the response of the vestibular receptors during motion deceleration in a given direction is similar to the response evoked when accelerating in the opposite direction. Based on these physiological characteristics of the vestibular system, one would expect individuals to perceive their displacements as being shorter during the deceleration compared to the acceleration, and therefore underestimate the magnitude of their total displacements. In fact, several studies have shown that subjects tend to underestimate their passive body displacement in darkness, when the absence of feedback on performance prevents any learning (i.e., Bloomberg et al., 1991; Israël, 1992; Israël et al., 1993b; Blouin et al., 1995a,b, 1997; Quarck et al., 2009; Simoneau et al., 2009; Vidal and Bülthoff, 2010). Moreover, marked underestimation of body displacement during deceleration has already been reported (Guillaud et al., 2006). In this latter study, subjects were required to maintain the orientation of their arm stretched horizontal during passive body rotations at different frequencies (from 0.05 to 0.34 Hz). The subjects produced accurate compensatory arm movements in both the acceleration and deceleration phases of the rotation when visual information was available. However, when the angular displacements could be detected only through somatosensory and vestibular cues issued during chair rotation (i.e., condition without vision), the online compensatory arm movements remained accurate during the acceleration, but largely underestimated the rotation during the deceleration. These underestimations suggested that the vestibular system provided underrated information of body rotation during deceleration. On the other hand, using a more cognitive task, Mackrous and Simoneau (2011) found evidence that participants overestimate their angular displacements during whole body rotation accelerations. In their study, participants were asked to press a push button when they felt that their body’s midline had crossed a memorized target initially presented 60° in their periphery. The participants usually pressed the button near the end of the acceleration while they were still far from the target (on average 16°). In the present study, the subjects’ perception of body motion was assessed separately during the acceleration and deceleration phases of discrete body rotations to directly compare the cognitive estimate of body motion between these two phases.

## MATERIALS AND METHODS

Thirteen healthy adults (six females, seven males; mean age 32 years) volunteered to participate to the study. Prior to their engagement in the experiment, participants were asked if they were aware of current or past existence of any history of vestibular or other neurological disorders. Only those individuals without such history of disorders were selected. The experiment was conducted with the understanding and written consent of each participant, in accordance with the ethical standards of Aix-Marseille University and those set out in the 1964 Declaration of Helsinki.

A schematic representation of the experimental set-up is shown in **Figure 1**. The participants were seated on a chair positioned above the axis of a revolving platform placed in the middle of a closed room (2.4 × 2.4 m). The lights of the room were turned off during the experiment leaving the participants in complete darkness. The participants were secured on the chair with a three-point safety belt. They wore audio earphones diffusing a white noise to mask possible auditory spatial reference cues. The use of a neck brace helped to restrict head-on-trunk displacement. The platform was rotated by a servomotor controlled by a Smart Motor Control Card (Baldor Electric Company). During rotation, participants were required to fixate a LED attached to the chair, positioned approximately 1 m in front of them. This procedure was used to minimize eye movements and to keep gaze direction similar across participants. This was judged important because it has been reported that different gaze direction may lead to different perception of rotations (Quarck et al., 2009). The participants’ task was to report online the perceived rotation by rotating the manipulandum’s handle. This handle was mounted on the axis of a potentiometer incorporated onto a small box positioned on the chair in front of them. The participants were told to rotate the handle by the same angular amplitude as the chair and in the same direction. They did not receive feedback about their performance during the experiment. Note that this method differs from the continuous pointing task in which participants must rotate the arm or pointer in the opposite direction of the rotations. Because vestibulomotor transformations are generally employed to stabilize the body and its segments (e.g., eyes, arm) during motion, movements of the handle in the present experimental task were more likely to arise from vestibular-issued cognitive processes than from more direct vestibulomotor processes. Rotations of the handle were measured with the potentiometer and the platform’s angular position was returned to the computer by the axis control card. Signals sent to the chair, and received from the manipulandum and chair, were handled by a 12-bit analog/digital board (Keithley Instruments Inc.) installed in a real-time controller system (ADwin Pro, Jäger Computergesteuerte Messtechnik), which was programmed using custom-designed software (Docometre). The manipulandum potentiometer input was sampled at 500 Hz. During data reduction, both the handle and chair position signals were filtered using a zero-phase low-pass digital filter set at 10 Hz. Eye movements were monitored (but not recorded) to ensure that participants fixated the LED in front of them during the trials. To do so, we used a pair of goggles with a custom video-oculography unit (not represented in **Figure 1**) placed in front of the non-dominant, left eye (determined by the hole-in-card test; Miles, 1930). During the trials, the experimenter verified whether participants complied with the instruction regarding fixation of the chair-fixed LED. No trials had to be rejected based on non-compliance with this instruction.

**FIGURE 2:**
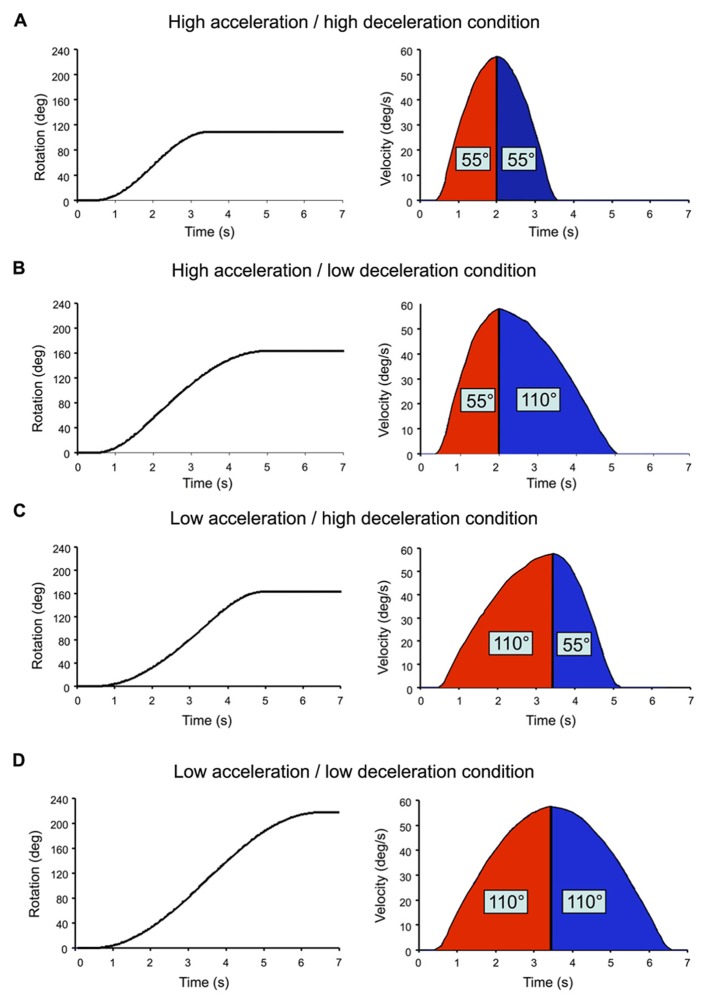
**Angular displacement (returned to the computer by the axis control card) and velocity (computed offline) of the chair in each tested combination of acceleration and decele-ration.** Rotation onset occurred 500 ms after the onset of the recordings. **(A)** High acceleration/high deceleration condition; **(B**) high acceleration/low deceleration condition; **(C)** low acceleration/high deceleration condition; **(D)** low acceleration/low deceleration condition.

The chair rotations were sinusoid-type angular velocity profiles, which had similar peak amplitude of 57°/s but varied in rise times (**Figure 2**). Acceleration duration (i.e., time to peak velocity) and deceleration duration (i.e., time between peak velocity and rotation offset) were either 1.5 or 3 s. High acceleration or deceleration (AccHigh; DecHigh) will refer to those that lasted 1.5 s (peak of 60°/s^2^) and low acceleration or deceleration (AccLow; DecLow) to those lasting 3 s (peak of 33°/s^2^). All combinations of acceleration and deceleration (*N* = 4) were used for both clockwise and counter-clockwise rotations. Depending on the acceleration and deceleration combination, the participants could be rotated by 110° (**Figure 2D**), 165° (**Figures 2B,C**) or 220° (**Figure 2A**). We used different combinations of acceleration and deceleration in order to diminish the participants’ possibility to predict the deceleration on the basis of the previous acceleration. By doing so, it was also possible to test whether changing acceleration and deceleration intensities had an effect of the participants’ perception of their rotations. The position reached by the chair at the end of the trials was used as the starting position of the next trial. At least 25 s separated each trial. Eight trials were run per condition and the different conditions were randomly presented.

**FIGURE 3:**
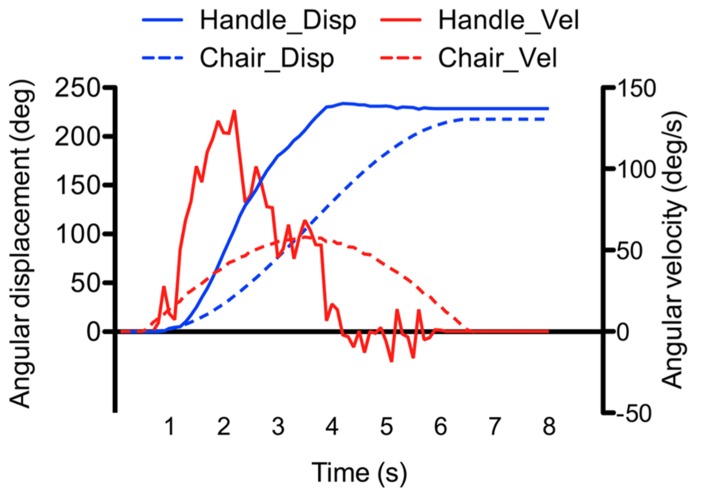
**Example of recorded angular displacement and velocity of the chair and manipulandumss handle (the example was taken from the AccLow-DecLow condition which rotated the participant by 220°)**.

**Figure 3** shows one example of the angular displacements and velocities of the chair and manipulandum’s handle (i.e., participant’s response). As depicted, the angular displacements of the handle increased relatively smoothly during the rotation. Clearly however, the velocity of the handle and of the chair motions did not match (compare velocity traces of **Figure 3**). The fact that the participants were instructed to reproduce their perceived angular displacements rather then the velocity of their rotations could explain the large discrepancy between the chair and handle velocity measurements (note that the velocity reproduction was also less reliable than in continuous pointing tasks, e.g., Ivanenko et al., 1997a; Guillaud et al., 2006). To assess participants’ perception of rotations, we computed the ratio between the amplitude of the perceived rotations (as measured by the rotating handle) and the amplitude of the actual chair rotations. This ratio, hereafter referred to as the gain, was measured at the end of the rotations (total gain), and was also computed separately for the acceleration and deceleration phases. Unless otherwise specified, the computed gains were analyzed using separate ANOVAs. First, the effects of the phase, intensity and direction were specifically tested using a 2 (Phase: acceleration, deceleration) × 2 (Intensity: low, high) × 2 (Direction: counter-clockwise, clockwise) repeated measures ANOVAs. Then, the gain measured at the end of the rotation (total gain) was submitted to a 4 (Profile: AccLow-DecLow; AccLow-DecHigh; AccHigh-DecLow; AccHigh-DecHigh) × 2 (Direction: counter-clockwise, clockwise) repeated measures ANOVA. The same analyzes were also performed on the variability of the gains (i.e., intra-subject standard deviation of the mean). The statistical threshold was set to *p* < 0.05 for all statistical analyzes and significant effects were further analyzed using Newman–Keuls tests. All significant effects and interactions are reported.

## RESULTS

**FIGURE 4:**
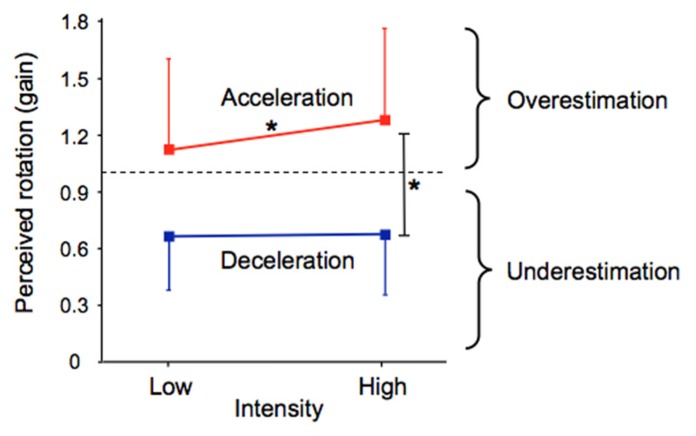
**Mean acceleration and deceleration gains as a function of their intensity.** The vertical bars represent between-subjects standard deviations. *p < 0.01.

The participants’ perception of their passive displacements markedly differed between the acceleration and deceleration phases of the rotation and was also dependent on the intensity of these phases (see **Figure 4**). The effects of the independent variables on the gain were confirmed by the 2 (Phase) × 2 (Intensity) × 2 (Direction) ANOVA which revealed a significant main effect of Phase (*F*(1,12) = 50.83, *p* < 0.001) and a significant Phase × Intensity interaction (*F*(1,12) = 8.18, *p* < 0.01). On average, the gain was markedly greater during the acceleration (mean = 1.19) than during the deceleration (mean = 0.67). The breakdown of the interaction revealed that the factor Intensity had a significant effect only during the acceleration, the gain being greater in the high (mean = 1.28) than in the low (mean = 1.11) intensity. As shown by the size of the vertical bars in **Figure 4**, the gain computed in the different phases of the rotation considerably varied between the participants.

The same 2 × 2 × 2 ANOVA performed on the intra-subject variability revealed significant effects of Phase (*F*(1,12) = 15.56, *p* < 0.01) and Intensity (*F*(1,12) = 33.54, *p* < 0.001; not illustrated). Participants showed greater variability in perceiving their rotation during the acceleration phase (mean = 0.23) than during the deceleration phase (mean = 0.17). High intensity rotational stimuli (mean = 0.23) also resulted in greater variability compared to low intensity (mean = 0.17). Therefore, the greater mean gains were associated with the greater intra-subject variability.

**FIGURE 5:**
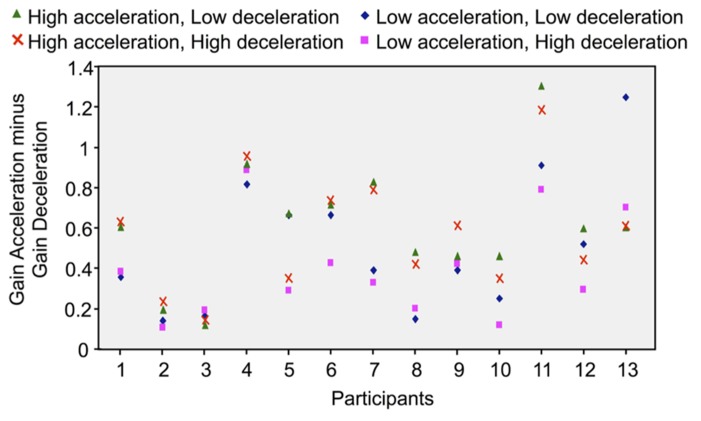
**Difference between the acceleration and deceleration gains computed in each rotational stimulus and for each participant (the positive values indicate that the gains were greater in the acceleration than in the deceleration)**.

Results reported above showed considerable inter-participant variability in the perception of self-motion, irrespectively of the considered phase and intensity of the rotation. To determine whether the effect of Phase (i.e., greater gain in acceleration than in deceleration) was consistent across participants, we subtracted the deceleration’s gain from the acceleration’s gain, for each type of velocity profile (averaged for counter-clockwise and clockwise rotations) and each participant. Positive differences resulting from this computation would indicate greater gain in the acceleration than in the deceleration. As shown by **Figure 5**, the differences between the gains of both rotation phases considerably varied between participants. But more importantly, only positive differences resulted from this computation, which indicates that the gain was greater in the acceleration than in the deceleration for all participants and for all combinations of acceleration and deceleration. It is worth noting that participants’ rotatory response may have lagged the chair rotation due to delays in sensorimotor systems and to the time required to cognitively process the vestibular input (see Barnett-Cowan, 2013 for a review). As such, because we measured acceleration gain at the end of the acceleration without correcting for a possible lag, it must be noted that the high acceleration gain computed here, which supports Mackrous and Simoneau’s (2011) findings, could actually underestimate the actual participants’ perception of their displacement during that phase.

**FIGURE 6:**
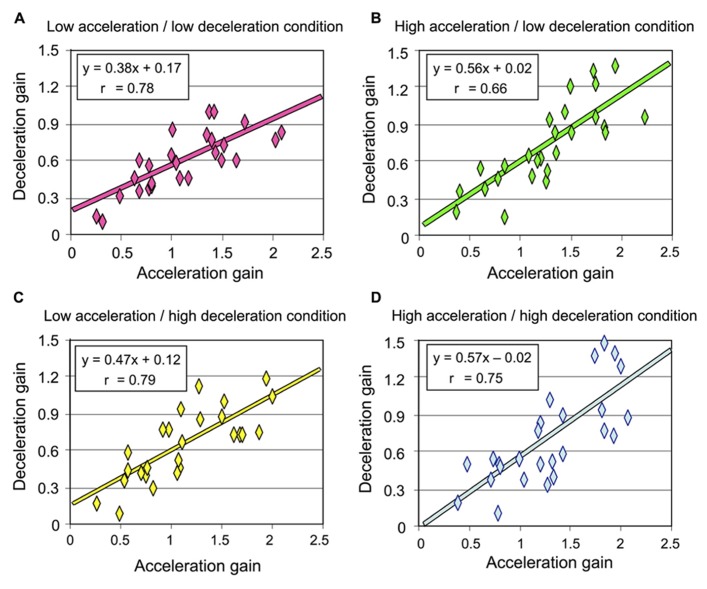
**Deceleration gain of all participants plotted against the acceleration in each rotational stimulus (combining counter-clockwise and clockwise rotations) (A) Low acceleration/low deceleration condition; **(B)** high acceleration/low deceleration condition; **(C)** low acceleration/high deceleration condition; **(D)** high acceleration/high deceleration condition**.

As detailed above and illustrated in **Figure 4**, deceleration intensity had no significant effect on the participants’ perception of their rotation during that phase. We performed an additional analysis to specifically test whether the participants’ perception during the deceleration was influenced by the intensity of the preceding acceleration. To this end, we plotted the deceleration gain as a function of the acceleration gain for all participants and then computed the linear regression for each rotational stimulus (combining counter-clockwise and clockwise rotations). Our reasoning was as follows: if the intensity of the acceleration has an effect on the perception of rotation during the deceleration, then the slope of the linear regression should differ between the conditions with different acceleration intensities. The results of these regression analyzes are shown in **Figure 6**. In all conditions, the slope of the regression line was much smaller than 1, corroborating our previous analyzes showing that the gain during the deceleration was smaller than during the acceleration. However, the regressions computed in both conditions with high acceleration were characterized with larger slopes (mean 0.565, **Figures 6B,D**) than those computed in both conditions with low acceleration (mean 0.425, **Figures 6A,C**). Therefore, the greater was the acceleration, the greater was the perceived displacement during the deceleration. These results then suggest that the intensity of the angular acceleration influenced the participants’ perception of rotation during the subsequent decelerating phase.

**FIGURE 7:**
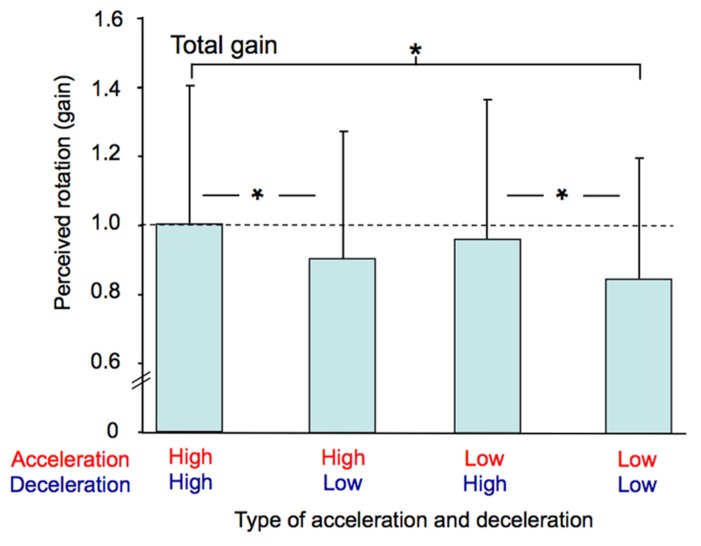
**Mean total gain computed for each combination of acceleration and deceleration (averaged for both counter-clockwise and clockwise rotations).** The vertical bars represent between-subjects standard deviations and the dotted line indicates a gain of one. * *p* < 0.01.

The participants’ perception of their total passive displacements also depended on the acceleration and deceleration with which they were rotated. The mean total gain (i.e., computed at the end of the rotation) was greatest in the AccHigh-DecHigh condition (mean = 0.99) and smallest in the AccLow-DecLow condition (mean = 0.85). The ANOVA performed on this gain revealed a significant effect of Profile (*F*(3,36) = 8.94, *p* < 0.001). As illustrated in **Figure 7** and confirmed by *post hoc* analyzes, the total gain in the AccHigh-DecHigh condition was significantly greater than in both the AccHigh-DecLow and AccLow-DecLow conditions and it was greater in the AccLow-DecHigh condition than in the AccLow-DecLow condition. The total gain considerably varied between the participants. This large variability is evident in **Figure 7** in which the errors bars represent between-subjects standard deviations, which ranged between 0.34 and 0.40 across conditions.

The ANOVA performed on the intra-subject variability of the total gain (not illustrated) also revealed a significant effect of Profile (*F*(3,36) = 3.78, *p* < 0.05). *Post hoc* analyzes showed that this variability was greater in the AccHigh-DecHigh condition (mean = 0.17) than in the AccLow-DecLow condition (mean = 0.11). Intra-subject variability was therefore greater in the condition with the greatest total mean gain than in the condition with the smallest total mean gain.

## DISCUSSION

The results of the present study showed that the perception of self-motion greatly varies during the course of discrete body rotations. Indeed, we found that the participants’ estimates of their angular displacement were markedly larger during the acceleration phase of the rotation than during the deceleration phase. This was true even in conditions where the deceleration was the inverse mirror image of the acceleration, i.e., when the dynamics of the angular motion and the distance covered by the participants were similar in both phases.

In this study, we used a protocol that maximized the need of processing vestibular information online by asking the participants to report their perceived body angular displacements in real-time and making a random use of symmetric and asymmetric acceleration-deceleration profiles, which had different magnitudes and directions. This important aspect of the present study rendered predictive mechanisms inefficient to determine the actual body displacement. Prediction of motion, which is enhanced during symmetric acceleration-deceleration, is known to benefit to the online control of manual and ocular tracking of targets moving with such profiles (Poulton, 1981; Bahill and McDonald, 1983; Vercher and Gauthier, 1992; Xia and Barnes, 1999). With symmetric kinematic profiles, predictive mechanisms could particularly affect motion perception during deceleration, because the dynamics of the deceleration could be inferred from the dynamics of the acceleration.

Despite our methodological precaution of trial randomization, we cannot fully exclude a possible contribution of predictive mechanisms. Nevertheless, the regression lines computed after plotting the acceleration and deceleration gains can help to argue that in the context of the present experiment, acceleration-based prediction of body rotation during the deceleration may have been limited, at least in conditions with high acceleration. Before going into more detail, it appears useful to recall two important methodological aspects of the present experiment: (i) the gains reported here were measured using angular displacement data recorded from both the chair and the manipulandum (the latter being thought to reflect the participants’ perception of their own motion) and (ii) the chair angular displacement was smaller during high acceleration or deceleration (i.e., 55°) than during low acceleration or deceleration (i.e., 110°). Presumably, if the perceived deceleration were to be based on acceleration-derived prediction mechanisms, the slope of the regression line should have been smaller in the high acceleration/low deceleration condition than in the high acceleration/high deceleration condition. Indeed, predicting a 55° rotation during the deceleration in the former condition would have largely decreased the gain measured during the 110° deceleration. However, we found that the slopes computed in these conditions, which had similar acceleration (i.e., high) but different deceleration intensities were remarkably alike (compare **Figures 6B,D**) thus suggesting small impact of predictive mechanisms in the perception of body rotation during deceleration.

However, body acceleration may have affected the participants’ perception of self-motion during deceleration in a somewhat more indirect way, and that is, through the so-called velocity storage mechanism. Indeed, the brain is believed to be equipped with neural integrators that allow prolonging vestibular signals and therefore sensation of rotation, which cannot be explained on the sole basis of the labyrinths’ output (Raphan et al., 1979). For instance, this central process is thought to be responsible for the fact that participants perceive their angular velocity as increasing when it actually reaches a plateau (Brown, 1966; Sinha et al., 2008; Shaikh et al., 2013). These observations raise the question of the role that might play the accumulation of these neural integrators (velocity storage) during body acceleration in the perception of self-displacement during deceleration. In the present study, participants perceived greater rotation during the deceleration when the preceding acceleration was greater (66 vs. 30°/s^2^) and shorter (1.5 vs. 3 s). This was observed for both deceleration intensities (i.e., low, high). While the present psychophysical experiment does not allow one to make decisive claims regarding the neural mechanisms responsible for this finding, it appears reasonable to speculate greater velocity storage in the condition with greater angular acceleration, increasing the perceived angular displacement during their following deceleration.

To some extent, both the smaller gain observed in conditions with longer acceleration and the underestimation of the rotation observed during body deceleration could be linked to the effect of the turning stimuli on the relative motion between the endolymph and the semicircular canals. For instance, during clockwise head angular acceleration, due to the inertia of the endolymph, the hair bundles bend toward the kinocilium of the right labyrinth and away from the kinocilium of the left labyrinth. This leads to an increase and decrease discharge rate of the right and left vestibular afferent fibres, respectively (hence indicating clockwise head motion; Goldberg and Fernandez, 1971). Rapidly however, due to friction with the canals’ inner walls, the velocity of the endolymph approaches that of the head (Dodge, 1923; Goldberg and Fernandez, 1971; Laurens and Angelaki, 2011). It turns out that the rotation-related vestibular output decreases as the duration of the acceleration increases. In the present experiment, this phenomenon could explain the smaller gain observed when the acceleration lasted 3 s compared to 1.5 s.

Moreover, because the endolymph is moving in space during prolonged acceleration, during head deceleration, motion of the endolymph (again because of its inertia) becomes greater than that of the head and of the hair bundles. This causes the endolymph to push the hair bundles in the opposite direction than at the onset of head rotation, even though the head is still rotating (i.e., decelerating) in the same direction. This phenomenon is responsible for the perception of body rotation in the opposite direction to the actual rotation, when the deceleration occurs after prolonged rotation at constant velocity (e.g., Bockisch and Haslwanter, 1997; St George et al., 2011). In our experiment, this phenomenon, even if it could have been compensated for to some extent by velocity-storage mechanisms, could have contributed to the weakened sense of rotation during the deceleration.

The different perception of body rotations during the acceleration and deceleration phases could also be linked to the different effects of these phases on time perception, as reported in several papers (e.g., Israël et al., 2004; Capelli and Israël, 2007; Binetti et al., 2010, 2013). These studies showed that one perceives the time as being faster during body acceleration and as being shorter during deceleration. Based on these findings, suggestion has been made that vestibular stimulation could increase the rhythm of internal clock pacemaker during acceleration and decrease it during deceleration. Therefore, accelerating and decelerating the body would not strictly differently affect vestibular-mediated actions (as evidenced here and in Guillaud et al.’s (2006) study) but would also impact non-spatial cognitive processes. In all cases, the reported observations could suggest increased and decreased states of arousal during acceleration and deceleration, respectively. This would be compatible with the idea that attentional resources are required to accurately monitor changes in body orientation through vestibular inputs (Yardley et al., 1999) and that arousal increases the speed of the internal pacemaker (Burle and Casini, 2001). In this framework, it is possible that the participants’ attentional level was greater at the beginning of the rotations, i.e., during the acceleration (and more particularly during the short acceleration where we found greater gain), than afterward during the deceleration. Note that other studies have revealed significant impact of vestibular stimulations on not explicitly spatial cognitive processes (e.g. bodily awareness and number generation; Lopez et al., 2010; Ferrè et al., 2013a,b).

Lastly, we found large intra- and inter-individual variability in the perception of body rotation. This phenomenon was observed irrespectively of the considered rotation phase (i.e., acceleration, deceleration, total rotation). One possible explanation for this observation is the use of linear acceleration profile of body rotation, which is known to be associated with larger inter- and intra-subject variability than step acceleration (i.e., sharp and short acceleration that is followed by a constant acceleration, Gianna et al., 1996). The large variabilities observed here may also reflect the difficulty of the perceptual system to have access to continuous positional information from velocity-related vestibular input. In the present experiment, participants were asked to reproduce online their perceived angular displacement rather than the velocity of the rotation. Because the semi-circular canals respond to angular acceleration, a double integration is needed to estimate body orientation during rotations. A first peripheral integration is carried out within the vestibular apparatus itself and the second is carried out in the central nervous system (Robinson, 1989; McFarland and Fuchs, 1992). When performed offline and with less time constraints than in our task (i.e., after body motion), such transformation of vestibular cues into position cues appears less noisy (e.g., when estimating the position held before being passively moved, Bloomberg et al., 1988; Israël, 1992; Israël et al., 1993a, 1999; Blouin et al., 1995a,b, 1997). This may also suggest that the more accurate and less variable hand or arm responses reported in continuous pointing tasks studies – when moving participants point toward a memorized Earth-fixed object (see introduction) – were essentially based upon vestibular-issued velocity cues rather than positional cues.

## Conflict of Interest Statement

The authors declare that the research was conducted in the absence of any commercial or financial relationships that could be construed as a potential conflict of interest.

## AUTHOR CONTRIBUTIONS

Luc Tremblay, Liliane Borel, Laurence Mouchnino, and Jean Blouin conceived the study. Dany Paleressompoulle built the apparatus. Luc Tremblay and Andrew Kennedy performed the data acquisition. Luc Tremblay, Andrew Kennedy, and Jean Blouin analyzed the data. Luc Tremblay, Liliane Borel, Laurence Mouchnino, and Jean Blouin evaluated the data. Luc Tremblay and Jean Blouin wrote the manuscript. Liliane Borel and Laurence Mouchnino improved the manuscript.
